# 

**Published:** 1888-12

**Authors:** 


### CONTENTS.

### *MISCELLANEOUS.*

| | PAGE |
|---|---|
| Editorial Notes, | 621 |
| The Dose. P. P. Wells, M. D., | 623 |
| How to Find the Drug. N. W. Vandenburg, M. D., | 632 |
| Typhoid Fever. George H. Clark, M. D., | 635 |
| A Criticism of Dr. Holmes. J. T. Kent, M. D., | 639 |
| Proceedings of the Syracuse Hahnemannian Club. Frederick Hooker, M. D., Secretary, | 641 |
| The "Homœopathic" Medical Society of Penna., | 654 |
| How to Help us, | 646 |
| A Proposed New Homœopathic Hospital, | 647 |
| Cases from Practice. B. Simmons, M. D., | 648 |
| Locomotor Ataxy.—A Clinical Case. Clarence Willard Butler, M. D., | 651 |
| Carcinoma. John V. Allen, M. D., | 656 |
| Clinical Report of Three Cases Treated by R. B. Johnstone, M. D., | 660 |
| A Centre Shot with Sulphur. S. L., | 664 |
| A Peculiar Case. Alice B. Campbell, M. D., | 665 |
| Kali Bichromicum. W. P. Wesselhœft, M. D., | 666 |

### *BOOK NOTICES AND REVIEWS.*

| | |
|---|---|
| Medical Diagnosis—A Manual of Clinical Methods. By J. Graham Brown, M. D., | 667 |
| Transactions of the Forty-first Session of the American Institute of Homœopathy, | 667 |
| Fox's Atlas of Skin Diseases, with Photographic Illustrations, | 667 |
| The Preferable Climate for Phthisis, | 667 |
| Montreal Tracts on Homœopathy, No. 5, | 668 |
| A Rejoinder to Dr. Hughes. By Prosper Bender, M. D., | 668 |

### *NOTES AND NOTICES.*

| | |
|---|---|
| Removals—Hahnemannian Monthly: For Sale—Dr. Lippe's Library —Dr. Faulkner's Visiting List - Otis Clapp & Son's Visiting List —A Journal of Ophthalmology, Otology, etc., | 668 |

